# Factors influencing adult and adolescent completion of treatment for late syphilis: a mixed methods systematic review

**DOI:** 10.2471/BLT.24.291684

**Published:** 2025-04-01

**Authors:** Rochelle Tobin, Meagan Roberts, Nang Nge Nge Phoo, Laurens Manning, Richard Norman, Elizabeth Eadie-Mirams, Thel Hla, Jason J Ong, Renee Carey, Jonine Jancey, Daniel Vujcich

**Affiliations:** a Faculty of Health Sciences, Curtin School of Population Health, Curtin University, Kent St, Bentley WA 6102, Australia.; b University of Western Australia, Perth, Australia.; c Telethon Kids Institute, Nedlands, Australia.; d Monash University, Melbourne, Australia.

## Abstract

**Objective:**

To identify factors influencing the completion of a three-dose course of weekly intramuscular benzathine penicillin G injections by adults and adolescents with syphilis of unknown duration or late syphilis.

**Methods:**

We searched medical literature databases for studies reporting on factors influencing treatment completion by patients with syphilis aged 10 years or older and studies involving health professionals administering syphilis treatment. Studies could use quantitative, qualitative or mixed methods approaches. We conducted a systematic review following the JBI Manual for Evidence Synthesis method.

**Findings:**

We identified 24 eligible studies, of which 20 (83%) were published in 2010 or later, 19 (79%) focused on pregnant women, seven (29%) were conducted in Brazil, six (25%) in the United States of America and three (12%) in China. Health-care system-related factors influencing the noncompletion of treatment included the limited supply of, and limited access to, medication and inadequate follow-up systems. Other common factors were patients presenting late to antenatal services and social and economic factors, such as transportation barriers and a low educational level.

**Conclusion:**

A comprehensive systems approach is needed to increase the treatment completion rate for syphilis of unknown duration and late syphilis. Health service interventions, such as improving patient management systems, should be supplemented by actions to address social inequalities and shortages in the supply of benzathine penicillin G. Research is needed to understand barriers to treatment completion in high-income countries and among priority groups, including Indigenous people and men who have sex with men.

## Résumé

**Objectif:**

Identifier les facteurs influençant l’achèvement d’un traitement de trois doses d’injections intramusculaires hebdomadaires de benzathine benzylpénicilline par des adultes et des adolescents atteints d’une syphilis de durée inconnue ou d’une syphilis tardive.

**Méthodes:**

Nous avons consulté des bases de données de la littérature médicale pour trouver des études portant sur les facteurs influençant l’achèvement du traitement par des patients atteints de syphilis et âgés de 10 ans ou plus, ainsi que des études portant sur des professionnels de la santé administrant un traitement contre la syphilis. Ces études pouvaient utiliser des approches quantitatives, qualitatives ou des méthodes mixtes. Nous avons procédé à un examen systématique en suivant la méthode «Manual for Evidence Synthesis» du Joanna Briggs Institute.

**Résultats:**

Nous avons identifié 24 études éligibles, dont 20 (83%) ont été publiées en 2010 ou par la suite, 19 (79%) portaient sur des femmes enceintes, sept (29%) ont été menées au Brésil, six (25%) aux États-Unis et trois (12%) en Chine. Les facteurs liés au système de soins de santé qui influencent l’inachèvement du traitement comprennent la fourniture limitée de médicaments et l’accès limité à ceux-ci, ainsi que des systèmes de suivi inadéquats. D’autres facteurs courants étaient la présentation tardive des patientes aux services prénatals et les facteurs sociaux et économiques, tels que les obstacles dans l’utilisation des transports et un faible niveau d’éducation.

**Conclusion:**

Une approche systémique globale s’impose pour augmenter le taux d’achèvement du traitement de la syphilis de durée inconnue et de la syphilis tardive. Les interventions des services de santé, telles que l’amélioration des systèmes de prise en charge des patients, doivent s’accompagner d’actions visant à remédier aux inégalités sociales et aux pénuries de benzathine benzylpénicilline. Des recherches sont nécessaires pour comprendre les obstacles à l’achèvement du traitement dans les pays à revenu élevé et parmi les groupes prioritaires, notamment les hommes ayant des relations sexuelles avec d’autres hommes et les populations autochtones.

## Resumen

**Objetivo:**

Identificar los factores que influyen en la finalización de un tratamiento de tres dosis semanales de penicilina G benzatina por vía intramuscular en personas adultas y adolescentes con sífilis de duración desconocida o sífilis tardía.

**Métodos:**

Se realizó una búsqueda en bases de datos de literatura médica de estudios que informaran sobre factores que influyen en la finalización del tratamiento en pacientes con sífilis de 10 años o más, así como en estudios que incluyeran a profesionales sanitarios encargados de administrar el tratamiento para la sífilis. Se incluyeron estudios con enfoques cuantitativos, cualitativos o de métodos mixtos. Se llevó a cabo una revisión sistemática siguiendo la metodología del *JBI Manual for Evidence Synthesis*.

**Resultados:**

Se identificaron 24 estudios que cumplían los criterios de inclusión, de los cuales 20 (83%) fueron publicados en 2010 o posteriormente, 19 (79%) se centraron en mujeres embarazadas, siete (29%) se realizaron en Brasil, seis (25%) en Estados Unidos de América y tres (12%) en China. Entre los factores relacionados con el sistema sanitario que influyeron en la no finalización del tratamiento se encontraron el suministro limitado y el acceso restringido a la medicación, así como sistemas de seguimiento inadecuados. Otros factores frecuentes fueron la asistencia tardía de las pacientes a los servicios prenatales y diversos determinantes sociales y económicos, como las barreras de transporte y el bajo nivel educativo.

**Conclusión:**

Es necesario adoptar un enfoque sistémico integral para aumentar la tasa de finalización del tratamiento de la sífilis de duración desconocida y la sífilis tardía. Las intervenciones en los servicios sanitarios, como la mejora de los sistemas de gestión de pacientes, deben complementarse con medidas orientadas a reducir las desigualdades sociales y a paliar la escasez de penicilina G benzatina. Se requiere investigación adicional para comprender las barreras a la finalización del tratamiento en países de ingresos altos y en grupos prioritarios, como los hombres que tienen relaciones sexuales con hombres y las poblaciones indígenas.

## ملخص

الغرض تحديد العوامل المؤثرة على إكمال البالغين والمراهقين المصابين بمرض الزهري غير معروف المدة أو الزهري المتأخر، لدورة علاجية أسبوعية ثلاثية الجرعات من حقن البنزاثين بنسلين ج العضلية.

الطريقة قمنا بالبحث في قواعد بيانات المنشورات الطبية عن الدراسات التي تتناول العوامل المؤثرة على إكمال علاج مرضى الزهري الذين تبلغ أعمارهم 10 سنوات فأكثر، والدراسات التي تشمل أخصائيين صحيين يقدمون علاجًا للزهري. يمكن للدراسات استخدام أساليب كمية أو نوعية أو طرق مختلطة. قم بإجراء مراجعة منهجية وفقًا لدليل JBI لطرق تجميع الأدلة.

النتائج قمنا بتحديد 24 دراسة مؤهلة، نُشرت منها 20 دراسة (%83) في عام 2010 أو لاحقًا، وركزت 19 دراسة (%79) على السيدات الحوامل، وأُجريت سبع دراسات (%29) في البرازيل، وست دراسات (%25) في الولايات المتحدة الأمريكية، وثلاث دراسات (%12) في الصين. تشمل العوامل المرتبطة بنظام الرعاية الصحية والتي تؤثر على عدم إكمال العلاج: الإمدادات المحدودة للأدوية، والحصول المحدود عليها، وعدم كفاية أنظمة المتابعة. ومن العوامل الشائعة الأخرى: تأخر المرضى في السعي لخدمات ما قبل الولادة، والعوامل الاجتماعية والاقتصادية، مثل صعوبة الانتقالات، وانخفاض المستوى التعليمي.

الاستنتاج هناك حاجة إلى أسلوب شامل للأنظمة لزيادة معدل إكمال العلاج لمرض الزهري غير معروف المدة، والزهري المتأخر. يجب استكمال تدخلات الخدمات الصحية، مثل تحسين أنظمة إدارة المرضى، بإجراءات لمعالجة أوجه عدم المساواة الاجتماعية، ونقص إمدادات البنزاثين بنسلين ج. هناك حاجة إلى إجراء أبحاث لفهم عوائق إكمال العلاج في الدول ذات الدخل المرتفع وبين الفئات ذات الأولوية، بما في ذلك الرجال الذين يمارسون الجنس مع الرجال والسكان الأصليين.

## 摘要

**目的:**

为了确定影响病情持续时间不明的或晚期成人和青少年梅毒患者的苄星青霉素 G 肌肉注射疗程（共三剂次，每周注射一次）完成率的因素。

**方法:**

我们检索了医学文献数据库，以查找报告了 10 岁及以上梅毒患者治疗完成率影响因素的研究以及涉及卫生专业人员实施梅毒治疗的研究。研究可以采用定量方法、定性方法或者混合方法。

**结果:**

我们根据《JBI 证据综合手册》(JBI Manual for Evidence Synthesis) 中的方法进行了系统评价。我们确定了 24 项符合条件的研究，其中 20 项（占 83%）发布于 2010 年或之后，19 项（占 79%）以孕妇为研究对象，7 项（占 29%）在巴西开展，6 项（占 25%）在美国开展，3 项（占 12%）在中国开展。与卫生保健系统有关的治疗未完成率影响因素包括药物补给受限、药物获取渠道受限以及追踪系统不完善。其他常见因素包括患者接受产检服务的时间较晚以及交通障碍和教育水平低下等社会经济因素。

**结论:**

必须采用全面的系统做法以提高病情持续时间不明的或晚期梅毒患者的治疗完成率。在实施医疗服务干预措施（例如改善患者管理系统）的同时必须采取相应行动以解决社会不平等和苄星青霉素 G 供应不足的问题。必须开展相关研究以了解影响高收入国家优先人群（包括男男性接触者和土著人民）的治疗完成率的障碍。

## Резюме

**Цель:**

Выявление факторов, которые влияют на завершение курса из трех еженедельных внутримышечных инъекций бензатина пенициллина G взрослыми и подростками, страдающими от сифилиса неизвестной давности или запущенной формы данного заболевания.

**Методы:**

Авторы провели поиск исследований в базах данных медицинской литературы, в которых сообщалось о факторах, влияющих на завершение курса лечения у пациентов с сифилисом длительностью 10 лет или более. Проводился также поиск исследований с участием врачей, проводящих лечение от сифилиса. В этих исследованиях могли использоваться количественные, качественные или смешанные методологические подходы. Авторы выполнили систематический обзор, следуя руководству JBI по синтезу доказательных данных.

**Результаты:**

Было обнаружено 24 исследования, удовлетворяющие условиям. Из них 20 (83 %) были опубликованы в 2010 году или позже, 19 работ (79 %) были посвящены беременным женщинам, семь (29 %) были проведены в Бразилии, шесть (25 %) – в Соединенных Штатах Америки, три (12 %) – в Китае. Факторы, связанные с системой здравоохранения и препятствующие завершению курса лечения, включали ограниченность поставок и доступа к лекарствам, а также недостаточно эффективные системы последующего контроля. К другим распространенным факторам относились позднее обращение пациенток в службы дородового ухода и социально-экономические факторы, такие как проблемы с транспортной доступностью и низкий уровень образования.

**Вывод:**

Необходим всеобъемлющий системный подход для того, чтобы повысить уровень завершенности лечения сифилиса неизвестной давности или запущенной формы сифилиса. Вмешательства в сфере здравоохранения, такие как улучшение систем ведения пациентов, следует подкреплять действиями, направленными на уменьшение социального неравенства и нехватки безантина пенициллина G. Требуются исследования, которые помогли бы определить факторы, препятствующие завершению курса лечения в странах с высоким уровнем дохода и среди приоритетных групп населения, включая мужчин, практикующих секс с мужчинами, и представителей коренных народов.

## Introduction

Globally, an estimated 49.7 million people were living with syphilis in 2019. Of the 209 253 cases reported in the United States of America in 2023, 98 791 (47.2%) were syphilis of unknown duration or late syphilis.^1^ Here, late syphilis is defined as late-stage (i.e. tertiary) syphilis or late latent syphilis, where at least 2 years have passed since infection. In 2022, the World Health Organization (WHO) set a target for the reduction in the number of new syphilis cases globally from 7.1 million in 2020 to 0.71 million in 2030.^2^ To achieve this target, a key strategic priority is to monitor the cascade of care to ensure that timely treatment is provided with a minimal loss to follow-up.^2^ For most cases, *WHO guidelines for the treatment of Treponema pallidum (syphilis)* recommend treating adults and adolescents with primary, secondary and early latent (asymptomatic) syphilis of less than 2 years’ duration by administering a single intramuscular injection of benzathine penicillin G (i.e. 2.4 million units). For syphilis of unknown duration and late syphilis, by contrast, a regimen of three intramuscular injections delivered at weekly intervals (i.e. 7.2 million units in total) is generally recommended.^3^ However, limited data are available on the use of specific regimens or on the duration of treatment for syphilis of unknown duration and late syphilis.^3^ Theoretically, longer treatment is required for late syphilis as the causative bacteria divide more slowly at that stage.^4^ For syphilis of unknown duration, longer administration ensures that treatment is adequate regardless of when the infection was acquired. Furthermore, serological cure rates for syphilis of unknown duration and late syphilis with one dose of benzathine penicillin G are reported to be low, ranging from 33% to 39%.^3^

There are wide variations in the proportion of people with syphilis of unknown duration or late syphilis who receive all three doses of benzathine penicillin G as prescribed. The proportion reported varies from 93.5% in a Thai antenatal clinic study and 86% in a Bolivian antenatal clinic study to 61–65% in South African antenatal clinic studies;^5^^–^^8^ 54% in a Peruvian study of transgender women and men who have sex with men; and 43% in the general population in one county in the United States.^9^^,^^10^ Noncompletion of the three-dose regimen increases the risk of serious sequelae, including complications that affect the musculoskeletal, cardiovascular and central nervous systems.^11^ In pregnant women with syphilis, incomplete treatment may result in stillbirth, miscarriage, birth defects, preterm birth or neonatal death.^11^^,^^12^

The reasons people start but do not complete syphilis treatment are not clear. A review showed that factors that influenced whether pregnant women and their partners completed syphilis treatment regimens included: (i) the level of engagement with antenatal care; (ii) proximity to a clinic; (iii) knowledge about, and attitudes to, sexually transmissible infections; (iv) perceptions of pain associated with treatment; (v) medication availability; and (vi) clinical protocols and practices.^13^ However, information is also needed on factors that influence treatment pathways in priority groups with a higher prevalence of syphilis, such as Indigenous populations and men who have sex with men.^14^^,^^15^

The previous search identified only one systematic review of factors associated with the completeness of, and adherence to, benzathine penicillin G injections in a broad population group.^16^ That review examined treatment for rheumatic fever and rheumatic heart disease and found that adherence to treatment pathways was affected by: (i) disease severity; (ii) the quality of patient–staff interactions; (iii) the health worker’s cultural competence; (iv) patients’ perceptions of the pain associated with, and the efficacy of, treatment; and (v) the accessibility of treatment services, such as the provision of home-based care. However, the review’s findings may not be relevant for the treatment of syphilis of unknown duration and late syphilis because it is recommended that benzathine penicillin G is administered every three to four weeks for several years for acute rheumatic fever and rheumatic heart disease, with the duration determined by a range of sociodemographic and clinical factors.^17^

The aim of our review was to identify factors that influence the completion of a three-dose course of intramuscular benzathine penicillin G injections for syphilis by adults and adolescents to improve the health-care services and interventions involved in the syphilis care cascade.

## Methods

We followed the JBI Manual for Evidence Synthesis method for systematic reviews and the preferred reporting items for systematic reviews and meta-analyses.^18^^,^^19^ The protocol is registered in the PROSPERO database (CRD42023406247).^20^ Our review included studies that reported data on factors influencing the completion of a regimen of three intramuscular, 2.4 million-unit doses of benzathine penicillin G delivered at weekly intervals. Studies involving patients with syphilis aged 10 years or older, and studies involving health workers administering syphilis treatment were eligible. We considered all quantitative, qualitative and mixed-method studies conducted since 1944, the year when penicillin use for late-stage syphilis was first documented.^21^ Studies that investigated only factors related to the commencement of benzathine penicillin G treatment were not eligible for inclusion. In addition, opinion pieces, commentaries, protocols, conference abstracts, systematic reviews and literature reviews were excluded. There were no geographical or language limitations.

We initially searched Medline® and CINAHL databases to identify relevant articles to inform the search strategy. Then, we developed search strategies, with the assistance of a research librarian, for the Medline®, Embase, Web of Science Core Collection, CINAHL and Google Scholar databases (Box 1). Searches were conducted on 28 and 29 March 2023 and updated on 21 February 2024 and again on 30 January 2025. We assessed the first 200 results on Google Scholar, as previously recommended.^22^ We imported all records identified into EndNote 20 (Clarivate, Philadelphia, United States) and removed duplicates using the Bramer method.^23^ The grey literature and reference lists of the studies included were not searched.

Box 1 **Search strategies, systematic review of factors influencing treatment completion for late syphilis, worldwide, 1989–2025**

**Medline**

1. syphili*.mp. or exp syphilis/
2. pallidum.mp.
3. 1 or 2
4. treatment.mp. or exp therapeutics/
5. penicillin.mp. or exp penicillins/
6. exp anti-bacterial agents/ad, dt [administration & dosage, drug therapy]
7. exp penicillin g benzathine/ or benzathine.mp.
8. regimen*.mp.
9. treatment*.mp.
10. 4 or 5 or 6 or 7 or 8 or 9
11. exp patient compliance/ or exp “treatment adherence and compliance”/
12. exp treatment refusal/
13. ((treat* or therap* or medicat* or antibiotic* or penicillin or benzathine) adj10 (commenc* or adher* or “non-adher*” or nonadher* or complian* or “non-complian*” or noncomplian* or complet* or “non-complet*” or noncomplet* or refus* or “follow-up” or incomplete or adequat* or inadequat*)).mp.
14. cascade.mp.
15. 11 or 12 or 13 or 14
16. 3 and 10 and 15
17. limit 16 to yr = ”1944 -current”

**Embase**

1. syphili*.mp. or exp syphilis/
2. pallidum.mp.
3. 1 or 2
4. penicillin.mp. or exp penicillin derivative/
5. exp benzathine/ or benzathine.mp. or exp benzathine penicillin/
6. treatment.mp.
7. regimen*.mp. or exp drug dose regimen/ or exp drug therapy/
8. 4 or 5 or 6 or 7
9. ((treat* or therap* or medicat* or antibiotic* or penicillin or benzathine) adj10 (commenc* or adher* or “non-adher*” or nonadher* or complian* or “non-complian*” or noncomplian* or complet* or “non-complet*” or noncomplet* or refus* or “follow-up” or incomplete or adequat* or inadequat*)).mp.
10. cascade.mp.
11. exp treatment refusal/
12. exp patient compliance/
13. 9 or 10 or 11 or 12
14. 3 and 8 and 13
15. limit 14 to yr = ”1944 -current”

**Web of Science Core Collection**

1. syphili*
2. pallidum
3. 1 or 2
4. treatment
5. penicillin*
6. benzathine
7. regimen*
8. 4 or 5 or 6 or 7
9. ((treat* or therap* or medicat* or antibiotic* or penicillin or benzathine) and (commenc* or adher* or “non-adher*” or nonadher* or complian* or “non-complian*” or noncomplian* or complet* or “non-complet*” or noncomplet* or refus* or “follow-up” or incomplete or adequat* or inadequat*))
10. 3 and 8 and 9
11. limit 10 to yr = ”1944 -current”

**CINAHL**

1. syphili* or mh syphilis
2. pallidum
3. 1 or 2
4. treatment
5. mh penicillins
6. benzathine
7. regimen*
8. 4 or 5 or 6 or 7
9. mh patient compliance or mh treatment withdrawal or mh treatment refusal
10. ((treat* or therap* or medicat* or antibiotic* or penicillin or benzathine) and (commenc* or adher* or “non-adher*” or nonadher* or complian* or “non-complian*” or noncomplian* or complet* or “non-complet*” or noncomplet* or refus* or “follow-up” or incomplete or adequat* or inadequat*))
11. cascade
12. 9 or 10 or 11
13. 3 and 8 and 12
14. limit 13 to yr = ”1944 -current”

**Google Scholar**

Search 1: penicillin syphilis adherence (Limits: title only and limit yr = ”1944 -current”)

Search 2: penicillin syphilis treatment (Limits: title only and limit yr = ”1944 -current”)

Notes: In Medline and Embase searches, mp. indicates multipurpose (e.g. title, abstract and keywords). In CINAHL searches, mh indicates both major and minor headings.

We imported study details into the Rayyan review management tool (Rayyan, Cambridge, United States).^24^ Non-English language studies were translated using Google Translate (Google Inc., Mountain View, United States) or through an accredited translation company. At least two members of a team of four reviewers screened study titles and abstracts against eligibility criteria, followed by full-text assessment of studies marked for possible inclusion. Any disagreements between reviewers were resolved by consensus following discussion; if a consensus could not be reached, a third team member made the final decision. Reasons for exclusion were recorded.

**Fig. 1:**
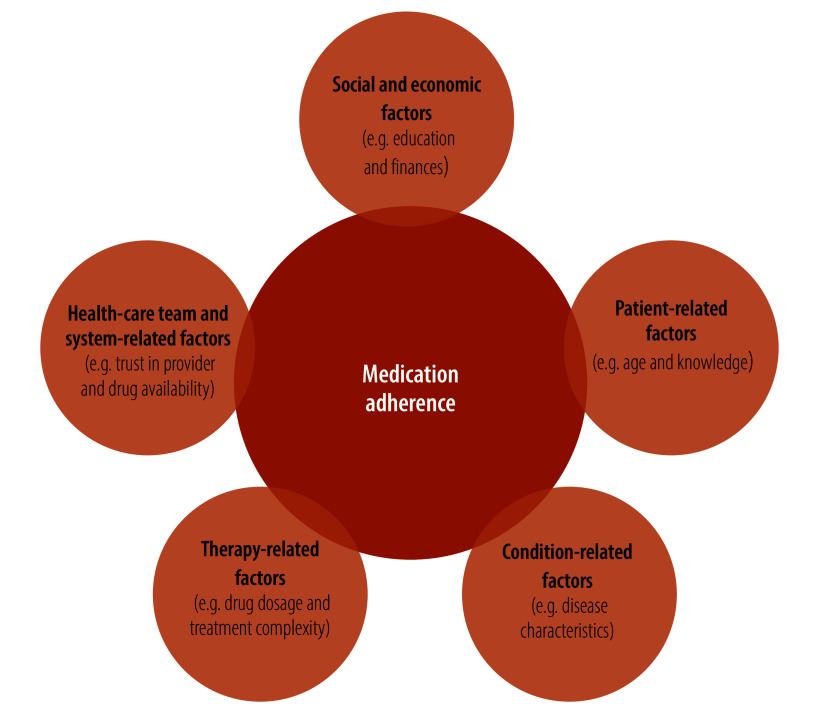
Dimensions of medication adherence, systematic review of factors influencing treatment completion for late syphilis, worldwide, 1989–2025

Note: The five dimensions of medication adherence are described in the World Health Organization’s publication *Adherence to long-term therapies: evidence for action*.^25^

We classified factors influencing treatment completion using WHO’s five dimensions of medication adherence,^25^ a widely used framework for characterizing adherence factors (Fig. 1).^26^

We also appraised all studies included in the systematic review for study quality using a checklist based on the Meta Quality Appraisal Tool (MetaQAT),^27^ which was developed to evaluate public health research across a variety of study designs. The tool assesses study quality using nine criteria in four domains: (i) relevancy (one criterion); (ii) reliability (three criteria); (iii) validity (four criteria); and (iv) applicability (one criterion).^27^ An overall judgment is produced for each criterion and domain. MetaQAT scores range from 0 to 18, with a high score indicating high quality: a score of 0 to 9 was classified as low quality, a score of 10 to 14 as medium quality and a score of 15 to 18 as high quality. For our review, this appraisal process demonstrated the relevance and appropriateness of the studies included for public health policy and practice. 

One author extracted following data from the included studies into Excel spreadsheets (Microsoft, Redmond, United States): (i) author or authors; (ii) year of publication or writing; (iii) publication journal; (iv) study design; (v) methodology (quantitative, qualitative or mixed-methods); (vi) methods; (vii) number of participants; (viii) participants’ characteristics; (ix) country in which the study was conducted; (x) health-care setting; (xi) study aim; and (xii) the type of factor reported to influence the completion of benzathine penicillin G treatment (i.e. condition-related factors, therapy-related factors, health-care system-related factors, patient-related factors, and social and economic factors). A second author crosschecked the extracted data and any disagreements were resolved by consensus. As our research question concerned the identification of factors influencing treatment completion rather than the measurement of effects, no meta-analysis was conducted.

## Results

**Fig. 2:**
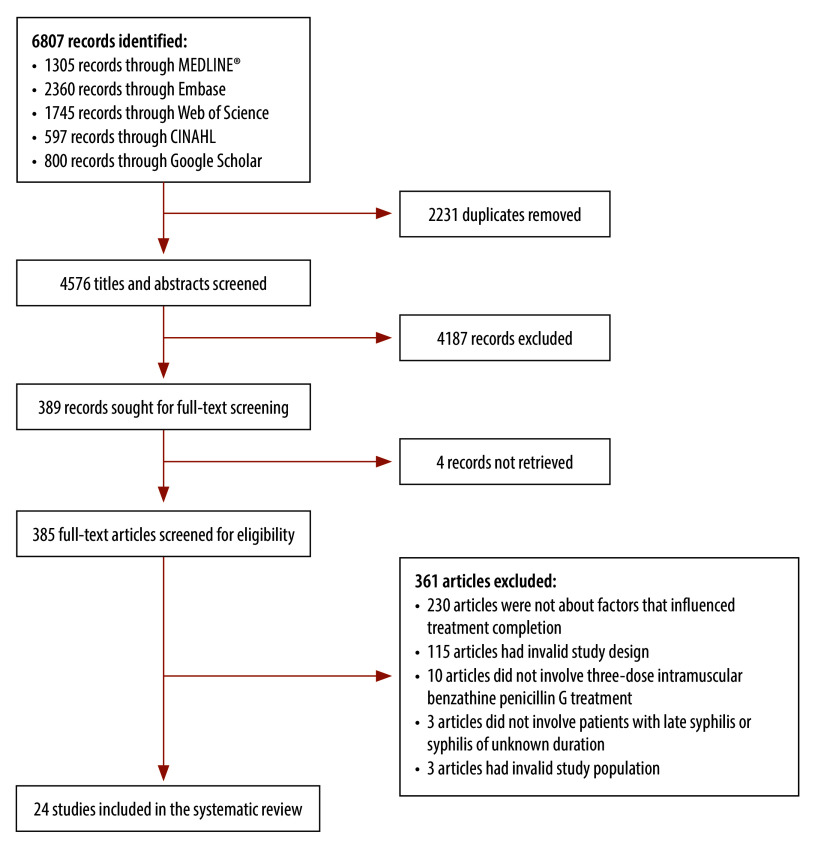
Study selection flowchart, systematic review of factors influencing treatment completion for late syphilis, worldwide, 1989–2025

We screened the titles and abstracts of 4576 studies, of which 389 were selected for full-text screening and 24 met the eligibility criteria (Fig. 2).^5^^–^^7^^,^^9^^,^^10^^,^^28^^–^^46^ The MetaQAT scores for these studies ranged from 9 to 17; 11 studies were categorized as high quality, 12 as medium quality and one as low quality. The MetaQAT appraisal revealed that many studies had treatment completion as a secondary or ancillary aim, which meant that these studies’ relevance to the aims of our review was not always clear. However, all 24 studies were relevant to public health policy and practice, and provided clear recommendations on the management and treatment of syphilis in primary care. As adequate treatment was not the primary focus of most studies, its definition varied. In addition, because a large proportion of studies examined treatment completion by pregnant women, incomplete treatment was frequently defined as treatment that was initiated less than 30 days before delivery.^10^^,^^28^^–^^35^ Several studies did not clearly describe what they defined as adequate treatment,^36^^–^^38^ with one study extending the definition of adequate treatment to include the partners of pregnant women.^35^ Most studies did not quantify the proportion of participants at each disease stage.^5^^–^^7^^,^^9^^,^^29^^–^^31^^,^^33^^,^^34^^,^^36^^–^^40^ Consequently, lack of clarity about the definition of adequate treatment, and about whether adequate treatment was the administration of one or three benzathine penicillin G doses, made it difficult to determine if the influencing factors reported in the studies related to completion of the three-dose treatment regimen or to another aspect of treatment, such as treatment uptake or, for pregnant women, treatment before delivery.

Table 1 (available at https://www.who.int/publications/journals/bulletin/) summarizes the main characteristics of the included studies. Most were published in 2010 or later (83%; 20)^6^^,^^9^^,^^10^^,^^28^^,^^30^^–^^32^^,^^34^^–^^46^ and were conducted in Brazil (29%; 7)^30^^,^^34^^,^^35^^,^^37^^,^^38^^,^^40^^,^^41^ or United States (25%; 6).^10^^,^^31^^,^^42^^,^^44^^–^^46^ The majority included only pregnant women (79%; 19/24).^5^^–^^7^^,^^28^^–^^37^^,^^39^^,^^41^^–^^44^^,^^46^ Of the 24 studies, two (8%) looked at treatment completion among gender- or sexually diverse people, specifically transgender women and men who have sex with men,^9^^,^^40^ and three (13%) investigated people with syphilis in the general population.^10^^,^^38^^,^^45^ Few studies collected data through surveys, interviews or focus groups (25%; 6);^29^^,^^33^^,^^36^^–^^38^^,^^40^ instead, most studies (71%; 17) involved the quantitative analysis of cross-sectional data from reviews of medical records or syphilis registers.^7^^,^^9^^,^^10^^,^^28^^,^^29^^,^^31^^–^^36^^,^^41^^–^^46^

**Table 1:** Study characteristics, systematic review of factors influencing treatment completion for late syphilis, worldwide, 1989–2025

Study authors and year of publication	Study aim	Study country	Study setting	Study type and methods	Data source and study participants	Stage of syphilis	Adequacy of treatment	Analysis of factors affecting treatment completion
Phaosavasdi et al. 1989^5^	To determine the effectiveness of the three-dose benzathine penicillin G regimen for the treatment of syphilis in pregnancy	Thailand	Tertiary care	Quantitative: matched cohort study	Antenatal records of 197 pregnant women with syphilis	ND	3.6% (7/197) of pregnant women received inadequate treatment of only one or two benzathine penicillin G injections	Descriptive analysis
Rutgers 1993^33^	To assess the quality of antenatal care regarding the detection and management of syphilis	Zimbabwe	Primary and tertiary care	Mixed methods: cross-sectional register review and interviews	Antenatal records of 1433 pregnant women	ND	ND	Descriptive analysis of qualitative findings
Beksinka et al. 2002^29^	To evaluate the process of providing routine syphilis screening for antenatal care clients	South Africa	Primary and tertiary care	Qualitative: interviews, focus groups, observations and inventory and client flow analysis	Key informant interviews with: (i) 9 policy-makers and clinic staff; (ii) 112 antenatal clinic clients (i.e. pregnant women); (iii) 22 postnatal clinic clients (i.e. postpartum women); and (iv) 16 antenatal clinic clients focus groups	ND	ND	Descriptive analysis of qualitative findings
Mullick et al. 2005^7^	To establish the degree of compliance with treatment for syphilis by pregnant women	South Africa	Tertiary care	Quantitative: cross-sectional medical record review	Antenatal records of 18 128 pregnant women with syphilis, of whom 188 tested positive for syphilis	ND	64.8% (122/188) of pregnant women received all three benzathine penicillin G doses, 5.8% (11/188) received two doses, 13.2% (23/188) received one dose and 15.9% (30/188) received none^a^	Frequency analysis
Campos et al. 2010^30^	To identify reasons for inadequate treatment of pregnant women	Brazil	Tertiary care	Quantitative: cross-sectional survey and medical record review	Medical records and questionnaires involving 58 pregnant women with syphilis	ND	94.8% (55/58) of pregnant women received inadequate treatment	Frequency analysis
Zhu et al. 2010^39^	To assess trends and determinants of maternal and congenital syphilis in Shanghai	China	Primary and tertiary care	Quantitative: prospective cohort	Database records of 1471 pregnant women with syphilis	ND	26.6% (392/1471) received incomplete treatment	Frequency analysis
Diaz Olavarrieta et al. 2011^6^	To assess the feasibility and acceptability of a patient-led syphilis partner notification strategy among pregnant women with syphilis and their male partners and to assess treatment completion	Bolivia (Plurinational State of)	Tertiary care	Quantitative: cross-sectional survey	Self-administered surveys by 144 pregnant women and 137 male partners	Due to the nature of the study and the test used (immuno-chromato-graphic strip), the stage could not be determined	86.1% (124/144) received three benzathine penicillin G doses, 11.8% (17/144) had incomplete treatment (i.e. one or two doses) and 2.1% (3/144) received treatment elsewhere	Descriptive and bivariate analysis
Tang et al. 2015^9^	To assess if men who have sex with men and transgender women who screen positive for syphilis are receiving appropriate care and treatment	Peru	Multi-city database	Quantitative: cross-sectional register and medical record review	Database records of 314 men who have sex with men and transgender women with syphilis, of whom 63 were prescribed three weekly doses of benzathine penicillin G	ND	46.0% (29/63) did not receive all three benzathine penicillin G doses	Frequency and regression analysis
Rodrigues et al. 2016^38^	To analyse the practice of nurses in primary health care monitoring syphilis	Brazil	Primary care	Qualitative: interviews	Interviews with 18 nurses	ND	ND	None
Nunes et al. 2017^37^	To identify difficulties found by professionals affecting the adherence to syphilis treatment by pregnant women and their partners	Brazil	Antenatal clinic (undefined)	Qualitative: interviews	Interviews with 4 nurses	ND	ND	ND
Santos et al. 2017^41^	To assess the knowledge and compliance of health professionals regarding diagnostic and treatment practices for syphilis in patients admitted for childbirth to public maternity hospitals	Brazil	Tertiary care	Quantitative: cross-sectional survey	Questionnaire with 159 obstetricians and maternity nurses	Not determined as the study investigated obstetricians’ and nurses’ knowledge	ND	Frequency analysis
Kidd et al. 2018^31^	To estimate the proportion of potential congenital syphilis cases averted with current prevention efforts and to develop a classification framework to better describe why reported cases were not averted	United States	Nationwide database	Quantitative: cross-sectional register review	Database records of 2508 pregnant women with syphilis	ND	76.9% (1928/2508) of pregnant women received adequate treatment and 7.6% (48/628) of mothers of reported congenital syphilis cases received adequate treatment	Frequency analysis
Zhang et al. 2018^43^	To describe the epidemiological characteristics of pregnant women with syphilis and to investigate the determinants of adverse pregnancy outcomes	China	City database	Quantitative: cross-sectional register review	Database records of 807 pregnant women with syphilis	Latent (89.3%; 721/807), primary (2.7%; 22/807), secondary (0.6%; 5/807), tertiary (0.2%; 2/807) and unknown duration (7.1%; 57/807)	40.5% (327/807) were inadequately treated	Frequency and regression analysis
Rahman et al. 2019^42^	To assess the impact of congenital syphilis case review boards on preventing future cases by examining case reviews, identifying preventable factors and evaluating changes in provider practices	United States	Congenital syphilis case review boards	Mixed methods: analysis of case review board records	Congenital syphilis case review board records of 79 congenital syphilis cases	Primary (3.8%; 3/79), secondary (15.2%; 12/79), early latent (36.7%; 29/79), high-titre late latent (17.7%; 14/79) and low-titre late latent (26.6%; 21/79)	ND	Frequency analysis
Anugulruengkitt et al. 2020^28^	To determine the rate of congenital syphilis and to identify gaps in prevention	Thailand	Tertiary care	Quantitative: cross-sectional medical record review	Medical records of 69 pregnant women and their 30 infants	Primary (0%; 0/69 of women), secondary (2.9%; 2/69), early latent (7.2%; 5/69), late latent or unknown duration (88.4%; 61/69) and neurosyphilis (1.5%; 1/69)	40.6% (28/69) of pregnant women received inadequate treatment	None
de Oliviera et al. 2020^35^	To analyse processes that trigger the vertical transmission of syphilis by reviewing gestational and congenital syphilis notifications	Brazil	City database	Quantitative: cross-sectional register review	Database records of 129 pregnant women with confirmed vertical syphilis transmission	Primary syphilis (51.2%; 66/129); other stages not determined	76.7% (99/129) of pregnant women received inadequate treatment	Factors were described in the discussion
Swayze et al. 2022^34^	To identify parameters associated with the mother-to-child transmission of syphilis by pregnant women	Brazil	Tertiary care	Quantitative: cross-sectional register review	Hospital records of 1541 pregnant women with syphilis	ND	60.6% (934/1541) of pregnant women received inadequate treatment, of whom 84.8% (792/934) were not treated while pregnant or had treatment initiated within 30 days of delivery because of a late diagnosis, 13.7% (128/934) were treated but titres did not decline or increased after treatment and no treatment was recorded for the remainder	Frequency and regression analysis
Delvaux et al. 2023^36^	To describe the challenges and outcomes of implementing a national syphilis follow-up system to improve syphilis management in maternal and child health services	Cambodia	Public health facilities	Mixed methods: register review, interviews and focus groups	Database records of 470 pregnant women (of whom 315 tested positive for syphilis), interviews with 16 pregnant women and focus groups with 37 health workers	ND	27.9% (88/315) of women were treated adequately with benzathine penicillin G, 50.1% (158/315) were treated with other drugs (i.e. erythromycin or cefixime), and 21.9% (69/315) received no or unknown treatment	Thematic content analysis
Liu et al. 2023^32^	To identify correlates of receiving no treatment or inadequate treatment among pregnant women with syphilis	China	Nationwide database	Quantitative: cross-sectional register review	Database records of 1248 pregnant women with syphilis	Latent (80.9%; 1 010/1248), primary, secondary or tertiary (6.0%; 75/1248) and unknown duration (13.1%; 163/1248)	30.4% (379/1248) of women received no treatment or inadequate treatment, including 29.9% (302/1010) with latent syphilis, 23.7% (18/76) with primary, secondary or tertiary syphilis and 36.2% (59/163) with syphilis of unknown duration	Frequency and regression analysis
Mangone et al. 2023^10^	To quantify treatment for people diagnosed with late latent and unknown duration syphilis	United States	County database	Quantitative: cross-sectional register review	Database records of 14 924 people with syphilis	Late latent or unknown duration (36.0%; 5 372/14 924)	42.9% (2 302/5372) of people with late latent syphilis or syphilis of unknown duration received three benzathine penicillin G doses	Frequency analysis
McDonald et al. 2023^44^	To identify and classify missed opportunities to prevent congenital syphilis	United States	Nationwide database	Quantitative: cross-sectional register review	Database records of 3761 congenital syphilis cases	All primary and secondary combined	39.7% (1 494/3761) of birth parents received inadequate treatment	Frequency analysis
Carreira et al. 2024^40^	To identify factors associated with treatment noncompletion among transgender women and transvestites	Brazil	Community	Quantitative: cross-sectional survey	Structured interviews with 1317 transgender women of whom 211 tested positive for syphilis	ND	38.9% (82/211) of transgender women did not start or complete treatment	Frequency analysis
Clarkson-During et al. 2024^45^	To examine demographic and clinical factors that may contribute to treatment noncompletion	United States	Tertiary care	Quantitative: medical record review	Medical record review of 171 syphilis patients	Primary (5.3%; 9/171), secondary (7.0%; 12/171), tertiary (6.4%; 11/171), early latent (5.3%; 9/171), and late or unknown (76.0%; 130/171)	48% (82/171) of patients did not complete treatment	Frequency analysis
Tannis et al. 2024^46^	To describe the characteristics associated with the lack of complete syphilis treatment during pregnancy	United States	Multi- jurisdictional database	Quantitative: medical record review	Surveillance data on syphilis in 1476 pregnant women from six United States’ jurisdictions	Primary, secondary or early latent (40.8%; 602/1476), late latent or unknown duration (57.6%; 850/1476), and other (1.6%; 24/1476)	19.8% (173/874) of people recommended three benzathine penicillin G doses were inadequately treated	Frequency and regression analysis

ND: not determined.

^a^ Details of the number of injections were missing for two records.

Fourteen studies (58%) reported the proportion of pregnant or postpartum women who commenced and completed syphilis treatment at least 30 days before delivery (Table 1). In these studies, the proportion of women who had no, inadequate or unknown treatment ranged from 4% to 88%. In 10 studies (42%), the proportion ranged from 41% to 88%.^6^^,^^28^^,^^30^^,^^32^^,^^34^^–^^36^^,^^43^^,^^44^^,^^46^ Because many of these studies involved women who had given birth to babies with congenital syphilis, the proportion who completed treatment was expected to be low. Of the five studies that did not investigate syphilis in pregnant women, two reported a treatment completion rate for the three-dose, benzathine penicillin G regimen. One study assessed treatment completion by people in the general population who had been diagnosed with late latent syphilis or syphilis of unknown duration, and found that only 43% ( 2302/5372) had received all three doses.^10^ Similarly, another study which investigated treatment completion by transgender women and men who have sex with men in Peru, found that 54% (34/63) received all three doses in the appropriate timeframe.^9^

Table 2 shows that the factors most often cited as influencing noncompletion of the three-dose, intramuscular, benzathine penicillin G treatment regimen were health-care system-related factors, patient-related factors, and social and economic factors. For the health-care system, frequently cited factors included: (i) the limited supply of, or access to, benzathine penicillin G;^10^^,^^29^^,^^34^^–^^37^^,^^41^ and (ii) an inadequate follow-up system for patients.^28^^,^^29^^,^^36^^,^^38^ One study highlighted the inadequate syphilis treatment women may receive during pregnancy when they attend several health-care services and when the transition between different health workers is not smooth.^34^ One study described how the availability of specialist health services for gender- and sexually diverse people could explain why transgender women were more likely to commence and complete treatment in one location compared to places without these services.^40^ The limited supply of, or access to, benzathine penicillin G was attributed to: (i) problems with national procurement processes;^36^ (ii) the health service’s inability to appropriately store the medication;^10^ and global supply shortages in 2014 and 2019 to 2020.^29^^,^^36^ The most common patient-related factor influencing treatment noncompletion was late presentation to antenatal services.^7^^,^^31^^,^^32^^,^^36^^,^^44^^,^^46^ One study reported that nearly half of patients who had inadequate treatment were diagnosed with syphilis only at delivery.^34^ Social and economic factors included: (i) barriers to transportation and the distance from health services;^10^^,^^36^^,^^42^ (ii) the lack of private health insurance;^45^ (iii) cultural or ethnic identity;^44^ (iv) residency status (e.g. migrants);^32^^,^^43^ (v) homelessness;^46^ (vi) substance use;^38^^,^^46^ (vii) a history of incarceration;^46^ and (viii) a low educational level.^34^^,^^39^

**Table 2:** Factors reported to influence treatment completion, by study, systematic review of factors influencing treatment completion for late syphilis, worldwide, 1989–2025

Study authors and year of publication	Factors influencing completion of benzathine penicillin G treatment for late syphilis				
	Condition-related factors	Therapy-related factors	Health-care team and system-related factors	Patient-related factors	Social and economic factors
Phaosavasdi et al. 1989^5^	NA	NA	Being required to undergo spinal puncture and staging of syphilis before treatment	NA	NA
Rutgers 1993^33^	NA	NA	Relationship between nurses and community enabler of treatment completion	NA	NA
Beksinka et al. 2002^29^	NA	NA	(i) Inadequate supply of, or access to, benzathine penicillin G; (ii) limited information provided to the patient on the importance of treatment compliance; (iii) inadequate follow-up system; and (iv) delays in pregnant women receiving syphilis test results, resulting in an inability to complete treatment before delivery	NA	NA
Mullick et al. 2005^7^	NA	NA	NA	Late presentation to antenatal services	NA
Campos et al. 2010^30^	NA	NA	Medical records incomplete or inaccessible during pregnancy	NA	NA
Zhu et al. 2010^39^	Abnormal reproductive history (not defined)	NA	NA	NA	Educational level of patient and partner
Diaz Olavarrieta et al. 2011^6^	NA	NA	NA	(i) Lack of time; and (ii) not notifying partners about diagnosis	NA
Tang et al. 2015^9^	NA	NA	NA	Patients who identified as transgender were more likely to be lost to follow-up	NA
Rodrigues et al. 2016^38^	NA	Lack of flexibility in treatment dosing	Inadequate follow-up systems due to lack of time and competing priorities	Lack of understanding of the need to return for further treatment (i.e. belief that one dose was adequate)	Substance use
Nunes et al. 2017^37^	NA	NA	(i) Inadequate supply of, or access to, benzathine penicillin G; and (ii) absence of syphilis treatment protocols	NA	NA
Santos et al. 2017^41^	NA	NA	(i) Inadequate supply of, or access to, benzathine penicillin G; and (ii) lack of knowledge of correct treatment regimen among health professionals (may be linked to staff turnover)	NA	NA
Kidd et al. 2018^31^	NA	NA	NA	Late presentation to antenatal services	NA
Zhang et al. 2018^43^	NA	NA	NA	NA	Barriers associated with being a migrant woman
Rahman et al. 2019^42^	NA	NA	NA	NA	Transportation barriers
Anugulruengkitt et al. 2020^28^	Recent infection near delivery	NA	(i) Inadequate follow-up system (e.g. no system to recall patients and loss to follow-up during referral or after treatment initiation); and (ii) improper treatment prescription	NA	NA
de Oliviera et al. 2020^35^	NA	NA	Inadequate supply of, or access to, benzathine penicillin G	NA	NA
Swayze et al. 2022^34^	Late diagnosis during pregnancy	NA	(i) Inadequate supply of, or access to, benzathine penicillin G; and (ii) ineffective system for enabling patient records to be accessed at different sites	Patient attendance at, and level of engagement with, antenatal care	Low educational level
Delvaux et al. 2023^36^	NA	NA	(i) Inadequate follow-up system, including lack of contact information; and (ii) inadequate supply of, or access to, benzathine penicillin G	(i) Late presentation to antenatal services; and (ii) behavioural or mental health issues	(i) Distance from health services; and (ii) patient’s financial challenges
Liu et al. 2023^32^	NA	NA	NA	(i) Women presenting and diagnosed late in pregnancy (unable to complete treatment before delivery); and (ii) migrant women who did not hold a residency permit	NA
Mangone et al. 2023^10^	NA	(i) Lack of flexibility in treatment dosing; and (ii) cost of treatment	(i) Inadequate supply of, or access to, benzathine penicillin G; and (ii) long waiting time for treatment	(i) Lack of time; and (ii) childcare obligations	Transportation barriers
McDonald et al. 2023^44^	NA	NA	NA	(i) No or no timely treatment during pregnancy; and (ii) patients who identified as non-Hispanic Black or African–American, and Hispanic and Latino patients	NA
Carreira et al. 2024^40^	NA	NA	Health services that specialize in sexual and gender diversity available	HIV-positive patients were more likely to complete treatment but being tested for an HIV infection was associated with a lower likelihood of treatment completion	Patients who experienced verbal assaults for being transgender were less likely to complete treatment
Clarkson-During et al. 2024^45^	NA	NA	(i) Patients diagnosed in the emergency department had the lowest rate of treatment completion, followed by those diagnosed in primary care; and (ii) patients with private health insurance were more likely to complete treatment	(i) Patients aged 40–49 years were least likely to complete treatment, whereas those aged 18–24 years were most likely; and (ii) heterosexual patients were less likely to complete treatment than those who did not identify as heterosexual	NA
Tannis et al. 2024^46^	Pregnancy outcome before 35 weeks	NA	NA	Receiving inappropriately timed or no prenatal care	(i) Lack of health insurance; (ii) history of incarceration; (iii) history of homelessness; and (iv) reported substance use during pregnancy

HIV: human immunodeficiency virus; NA: not applicable.

## Discussion

In this review, we found that the limited supply of, and limited access to, benzathine penicillin G were frequently cited as reasons people diagnosed with syphilis of unknown duration or late syphilis did not complete the prescribed treatment regimen.^10^^,^^29^^,^^34^^–^^37^^,^^41^ This finding is supported by a WHO-led survey that found that 5% of countries reported benzathine penicillin G being out of stock in 2019.^47^ In December 2024, the United States also reported shortages of the drug, which were attributed to reliance on a single supplier and to a rise in the incidence of syphilis.^48^^,^^49^

Currently, few manufacturers produce the active pharmaceutical ingredient in benzathine penicillin G because: (i) demand is low compared to similar pharmaceutical products in production; (ii) the range of applications is decreasing; (iii) there are competitive financial pressures; and (iv) profit margins are narrow.^49^ Regrettably, supply shortages have led to non-recommended treatments being used and to countries procuring benzathine penicillin G from unregulated suppliers.^48^ To increase the accuracy of predicted demand and procurement requirements for benzathine penicillin G, WHO has announced plans to collaborate with Member States to improve the supply chain infrastructure.^47^ Other recommended structural solutions include: (i) more extensive market interventions; (ii) improvements in drug quality assurance processes to reduce the risk of manufacturing suspensions by regulatory authorities; (iii) increasing the supply of benzathine penicillin G;^47^^,^^49^ and (iv) encouraging countries to adopt mitigation strategies suited to their own individual requirements.^49^

At both national and regional levels, effective syphilis management strategies can involve: (i) increasing testing for the disease; (ii) training and capacity-building for health workers; (iii) improving community education; (iv) developing new pharmacological interventions (e.g. a single subcutaneous infusion of high-dose benzathine penicillin G instead of three intramuscular doses); (v) promoting organizational collaborations; and (vi) making health system governance more effective to optimize service delivery and policy implementation.^50^^,^^51^ A scoping review of global policy responses to syphilis found that no country had developed a comprehensive response that would lead to the control or elimination of syphilis in all key population groups.^52^ This finding was attributed to insufficient epidemiological surveillance, to a lack of clarity in promoting equity of access to health services, and to inadequate financing for sustainable policies. We found that equity of access had a substantial impact on treatment completion: people who had a low level of education,^34^^,^^39^ geographical or transport barriers to accessing health services,^10^^,^^36^^,^^42^ or financial challenges were all less likely to complete treatment.^36^

To facilitate timely follow-up of patients who require repeat doses of benzathine penicillin G, primary and tertiary health services need to improve patient management systems and infrastructure. Follow-up could be improved by: (i) ensuring that the contact details of patients commencing treatment are up to date;^36^ (ii) creating mechanisms that enable clinical data to be accessed at different sites in situations where patients frequently move between services;^34^ (iii) using recall reminders;^30^ (iv) providing treatment in nonclinical settings (e.g. in sex-on-premises venues);^44^ (v) training clinicians to make referrals and notify partners;^44^ and (vi) ensuring staff are allocated sufficient time and resources to adequately engage in follow-up.^38^

Difficulties with follow-up could also be overcome by developing alternative treatment regimens. Researchers have suggested that completion rates of benzathine penicillin G treatment for rheumatic heart disease could be improved by developing a new formulation that addresses issues with the dosing interval, pain and the administration mechanism.^53^ Other researchers have hypothesized that a single high-dose subcutaneous benzathine penicillin G infusion could achieve pharmaco-equivalence, leading to reduced health-care visits, improved adherence and lower health-care costs.^54^ A clinical trial is underway to examine the effectiveness of this approach for the treatment of syphilis.^55^

Most studies identified were conducted in the Region of the Americas. However, only three studies were from the WHO African Region, despite this region having the highest age-standardized incidence rates of syphilis in the world.^56^ Interestingly, no study we identified reported that pain affected treatment completion. In contrast, pain is frequently reported as a barrier to the completion of treatment of rheumatic heart disease.^53^ Few included studies examined treatment completion from the patient’s perspective, which may explain our finding.

Most studies in our review investigated factors that influenced the completion of syphilis treatment among pregnant women, despite the rate of syphilis among men who have sex with men rising in high-income countries.^57^ As a result, many of the barriers to treatment completion we identified are similar to those reported in an integrative review of inadequate syphilis treatment during pregnancy (e.g. the quality of antenatal care and late presentation to antenatal care).^13^ Nevertheless, we found additional social and economic factors associated with incomplete treatment, such as geographical and transportation barriers to accessing health services and financial challenges. In addition, our review provides some insights into how health services could increase treatment completion rates.

One study examined treatment completion rates among men who have sex with men and transgender women who have sex with men.^9^ Another examined completion rates among transgender women.^40^ Both reported that the completion rate for three doses of benzathine penicillin G was low. Interestingly, a study of the general population found that people who did not identify themselves as heterosexual were more likely to complete treatment.^45^ We found no studies that examined other priority population groups, such as sex workers or the Indigenous populations of Australia, Canada and the United States.^58^ It is likely that the factors influencing treatment completion in pregnant women differ from those in other priority population groups. For instance, case reviews of congenital syphilis in Australia’s Aboriginal population note that many women avoid contact with health services due to experiences of racism, having children removed from their care, and complications associated with homelessness and an itinerant lifestyle.^59^ Additionally, researchers highlighted how treatment uptake and completion by gender- and sexually diverse people may be negatively affected by the experience of verbal abuse and a lack of access to health services.^40^ For men who have sex with men, the existence of discriminatory laws that criminalize same-sex relationships may create a barrier to seeking health care.^15^ Targeted research involving other priority populations is warranted to better understand whether tailored interventions could increase the likelihood of treatment completion.

One strength of our review is that we employed the MetaQAT tool, which was specifically developed for public health research, to assess the quality of the studies included.^27^ Our review has several limitations. For example, we did not search the grey literature, the number of studies included was relatively small, only one person reviewed methodological quality, and few studies investigated treatment completion in population groups other than pregnant women. However, most studies involved the quantitative analyses of data from medical records and syphilis registers. Few qualitative studies examined reasons for inadequate treatment from the patient’s perspective.^29^^,^^30^ Consequently, our findings are generally limited to simplified variables that mostly quantify health workers’ observations. Recently, there have been increasing calls for qualitative research frameworks to investigate patients’ attitudes and subjective treatment experiences, including their ideas about medication efficacy, their perceptions of risk and how competing priorities are navigated.^60^^,^^61^ There is, then, a need for more qualitative research into factors that influence treatment completion.

In conclusion, our review highlights the importance of a comprehensive systems approach to increasing the treatment completion rate for syphilis of unknown duration and late syphilis. In addition to health service interventions, such as improving patient management systems, shortages in the supply of benzathine penicillin G and social inequalities must also be addressed. Furthermore, our findings reveal the importance of expanding syphilis research to encompass a wider spectrum of priority groups with a higher prevalence of the disease. Expanding the population groups being studied and employing a variety of data collection methods will lead to more tailored treatment interventions for syphilis that can respond effectively to the evolving epidemiology of the disease.
